# Risk modeling in transcatheter aortic valve replacement remains unsolved: an external validation study in 2946 German patients

**DOI:** 10.1007/s00392-020-01731-9

**Published:** 2020-08-26

**Authors:** Georg Wolff, Jasmin Shamekhi, Baravan Al-Kassou, Noriaki Tabata, Claudio Parco, Kathrin Klein, Oliver Maier, Alexander Sedaghat, Amin Polzin, Atsushi Sugiura, Christian Jung, Eberhard Grube, Ralf Westenfeld, Andrea Icks, Tobias Zeus, Jan-Malte Sinning, Stephan Baldus, Georg Nickenig, Malte Kelm, Verena Veulemans

**Affiliations:** 1 Division of Cardiology, Pulmonology and Vascular Medicine, Department of Internal Medicine, Heinrich Heine University, Medical Faculty, Moorenstr. 5, 40225 Düsseldorf, Germany; 2 Department of Medicine II, Heart Center Bonn, University Hospital Bonn, Bonn, Germany; 3 Division of Cardiology, Pneumology, Angiology and Intensive Care, Department of Internal Medicine III, University of Cologne, Cologne, Germany; 4 Institute for Health Services Research and Health Economics, Centre for Health and Society, Medical Faculty, Heinrich-Heine-University, Düsseldorf, Germany; 5 Transregio 259: Aortic Diseases-Scientific Network of University Heart Centers in Düsseldorf/Bonn/Cologne, Düsseldorf/Bonn/Cologne, Germany

**Keywords:** TAVR, TAVI, Risk prediction, Mortality, EuroSCORE, GAVS

## Abstract

**Background:**

Surgical risk prediction models are routinely used to guide decision-making for transcatheter aortic valve replacement (TAVR). New and updated TAVR-specific models have been developed to improve risk stratification; however, the best option remains unknown.

**Objective:**

To perform a comparative validation study of six risk models for the prediction of 30-day mortality in TAVR

**Methods and results:**

A total of 2946 patients undergoing transfemoral (TF, *n* = 2625) or transapical (TA, *n* = 321) TAVR from 2008 to 2018 from the German Rhine Transregio Aortic Diseases cohort were included. Six surgical and TAVR-specific risk scoring models (LogES I, ES II, STS PROM, FRANCE-2, OBSERVANT, GAVS-II) were evaluated for the prediction of 30-day mortality. Observed 30-day mortality was 3.7% (TF 3.2%; TA 7.5%), mean 30-day mortality risk prediction varied from 5.8 ± 5.0% (OBSERVANT) to 23.4 ± 15.9% (LogES I). Discrimination performance (ROC analysis, *c*-indices) ranged from 0.60 (OBSERVANT) to 0.67 (STS PROM), without significant differences between models, between TF or TA approach or over time. STS PROM discriminated numerically best in TF TAVR (*c*-index 0.66; range of *c*-indices 0.60 to 0.66); performance was very similar in TA TAVR (LogES I, ES II, FRANCE-2 and GAVS-II all with *c*-index 0.67). Regarding calibration, all risk scoring models—especially LogES I—overestimated mortality risk, especially in high-risk patients.

**Conclusions:**

Surgical as well as TAVR-specific risk scoring models showed mediocre performance in prediction of 30-day mortality risk for TAVR in the German Rhine Transregio Aortic Diseases cohort. Development of new or updated risk models is necessary to improve risk stratification.

**Graphic abstract:**

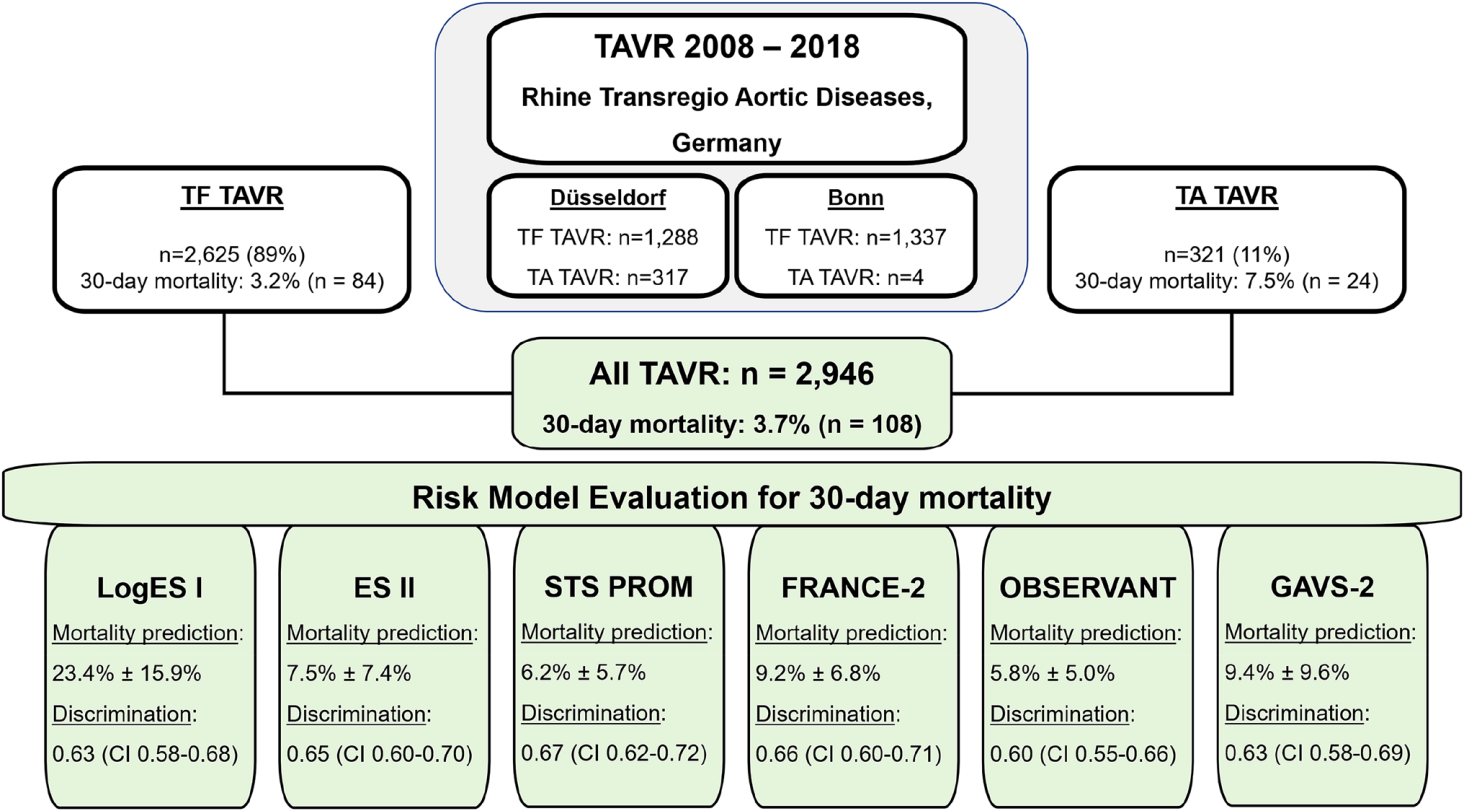

**Electronic supplementary material:**

The online version of this article (10.1007/s00392-020-01731-9) contains supplementary material, which is available to authorized users.

## Introduction

Transcatheter aortic valve replacement (TAVR) is the treatment option of choice in inoperable and high-risk patients with severe aortic valve stenosis [1, 2] and recently showed favorable outcomes also in intermediate-risk [3, 4] and low-risk patients [5, 6]. Patient and procedural characteristics determine risk for adverse clinical outcomes. Cardiovascular societies recommend clinical decision-making for TAVR within the Heart Team [1, 2], assisted by the use of statistical risk scoring models for individual patient risk stratification. Classical surgical risk models such as logistic EuroSCORE I (LogES I) [7, 8], its update EuroSCORE II (ES II) [9], or the Society of Thoracic Surgeons Predicted Risk of Mortality model (STS PROM) [10] are routinely used to gauge operative risk; however, they are known to overestimate mortality risk in TAVR [11–13]. Several TAVR-specific risk assessment models have been developed from national registry databases to optimize the predictive performance in the context of TAVR [14–16]. However, the optimal approach to risk prediction in TAVR still remains unknown, as their performance has been poor in validation studies [12, 17, 18].

We here aimed to evaluate the 30-day mortality prediction performance of six surgery- and TAVR-specific risk models (Table 1) in our German Rhine Transregio Aortic Diseases cohort of patients undergoing TAVR.

**Table 1:** Risk model characteristics

Risk model	Year of publication	Recruitment period	Recruitment region	Cohort size: development/validation (*n*)	Procedure characteristics	Discrimination performance (*c*-indices in validation cohort)
LogES I [7, 8]	1999/2003	1995	Europe	13,302/1479	Cardiac surgery	0.76
ES II [9]	2012	2010	Worldwide, predominantly Europe	16,828/5553	Cardiac surgery	0.81
STS PROM [10]	2009	2002–2006	US	65,855/43,904	Cardiac valve surgery	0.80
FRANCE-2 [15]	2014	2010–2011	France + Monaco	2552/1281	TAVR (TF 73.4%, TA 17.8%, other 8.8%)	0.59
OBSERVANT [14]	2014	2010–2012	Italy	1256/622	TAVR	0.71
GAVS-II [16]	2017	2011–2012	Germany	9027/9027	55% surgical AVR, 45% TAVR	0.74

Characteristics of the analyzed risk models for the prediction of 30-day mortality risk in TAVR

## Methods

### Study population and data collection

This study was performed as an all-comer analysis of patients treated with either TF or TA TAVR between 2008 and 2018 at the Heart Centers in Düsseldorf and Bonn, Germany, within the Rhine Transregio research consortium on aortic diseases. All patients were referred for TAVR procedures by local heart teams according to contemporary clinical practice and provided written informed consent for inclusion in prospective registries, with collection of clinical, procedural and follow-up data. The study was performed in accordance with the Declaration of Helsinki. The institutional Ethics Committee of the Heinrich-Heine University approved the study protocol (4080).

Clinical outcomes were systematically assessed using the Valve Academic Research Consortium consensus statement [19]. Primary clinical outcome of interest for risk model performance evaluation was 30-day mortality, secondary in-hospital outcomes are additionally reported.

### Statistics

Statistical and graphical data analysis was performed using Excel (Microsoft, USA), SPSS (version 23.0, SPSS Inc., Chicago, IL, USA), MedCalc 18.10 (MedCalc Software, Belgium) and GraphPad Prism (version 7.0, Graphpad Software, San Diego, CA, USA). Continuous data are described as means ± standard deviation (SD), ordinal/categorical data as counts and % of total, and receiver-operating-characteristic (ROC) curve analysis is summarized as c-indices (area-under-the-curve) with 95% confidence intervals (CI). Continuous variables were evaluated for normal distribution using the Kolmogorov–Smirnov test and compared with either Student’s *t* tests (for normally distributed samples) or Mann–Whitney-*U* tests; categorical data were compared with Chi-squared tests or Fisher’s exact tests (for expected values < 5); ROC curves were compared using a non-parametric approach according to DeLong et al. [20]. All statistical tests were two tailed and an α probability of *p* < 0.05 was considered statistically significant.

Six risk models for the prediction of 30-day mortality (Table 1: LogES I [7, 8], ES II [9], STS PROM [10], FRANCE-2 [15], OBSERVANT [14] and GAVS-II [16]) were calculated for individual patients. LogES I, ES II and STS were routinely available in the databases and used for heart team decision-making. FRANCE-2, OBSERVANT and GAVS-II were calculated as follows: (1) relevant variables (for details, see Supplementary material) were weighed according to model definitions; (2) weighed variables were transformed into 30-day mortality probability predictions according to model definitions. Missing values necessary for risk model calculation were imputed to the mean (continuous) or median (categorical) of the whole population. Risk model discrimination accuracy was evaluated using ROC analysis and the *c*-index [area-under-the-curve (AUC)] as a cumulative measure; risk model calibration accuracy/goodness-of-fit was evaluated by stratification of patients into risk quintiles and comparison of observed vs. expected events within risk strata; additionally, it was formally tested by calculation of a logistic regression model for 30-day mortality, with the score value as independent variable and the Hosmer–Lemeshow goodness-of-fit test [21]. Prespecified analyses of patients stratified by TF vs. TA approach, time period of the procedure and prosthetic valve device types were additionally performed to account for learning curves and technical developments.

## Results

### Patient population

A total of 2,946 patients underwent TAVR between 2008 and 2018 at the German Heart Centers of Düsseldorf (*n* = 1605) and Bonn (*n* = 1341) and were included in this analysis, with 2,625 TF TAVR (89%) and 321 TA TAVR (11%) procedures, respectively. Mean age was 80.9 ± 6.1 years (Suppl. Table 1), patients suffered from a high-cardiovascular-risk profile, including comorbidities of cardiovascular disease (67% coronary artery disease), previous cardiac interventional or surgical procedures (39% previous PCI, 15% previous CABG) and diabetes mellitus (30%). Characteristics differed significantly between patients treated with TF and TA TAVR procedures (Suppl. Table 1).

### Procedural characteristics across the TAVR era

TAVR developed from a rare procedure with high mortality (2008/2009: *n* = 61 with ~ 10% mortality) to a high-volume routine procedure (2018: *n* = 538 with 1.5% mortality) with low adverse events and shortened procedure duration (Table 2), which reflects a learning curve as well as continuous technical development. Patient age and estimated surgical mortality risk (LogES I) only declined marginally. TF TAVR became the primary access of choice (95.5% of procedures in 2018) over TA TAVR. The self-expandable Medtronic valves in different generations (early: CoreValve; newer generations: Evolut R and Evolut R Pro) were preferred to the balloon-expandable Edwards valves (early: Sapien XT; newer generation: Sapien 3) in TF TAVR; the latter were the primary choice in TA TAVR (Suppl. Table 1).

**Table 2:** Patient and procedural characteristics across the study period

Year	Age, years	LogES I	Patient count, *n* (%)	New gen. device, *n* (%)	TF/TA TAVR, *n/n* (% TF)	Procedure duration, min	Contrast volume, ml	30-Day mortality, *n* (%)
2008	84.5 ± 4.6	31.7 ± 17.6	17 (0.6)	0 (0)	17/0 (100)	59.1 ± 24.0	186.3 ± 45.8	1 (5.9)
2009	79.1 ± 6.7	27.5 ± 18.1	44 (1.5)	0 (0)	43/1 (97.7)	75.9 ± 29.1	217.1 ± 76.8	5 (11.4)
2010	82.5 ± 5.7	26.4 ± 14.5	78 (2.6)	0 (0)	60/18 (76.9)	82.6 ± 46.1	192.1 ± 80.9	5 (6.4)
2011	80.9 ± 6.3	23.2 ± 14.1	188 (6.4)	0 (0)	156/32 (83.0)	85.3 ± 39.0	166.9 ± 75.1	11 (5.9)
2012	81.1 ± 6.1	22.6 ± 14.2	118 (4.0)	0 (0)	88/30 (74.6)	88.6 ± 30.7	145.3 ± 88.6	9 (7.6)
2013	81.5 ± 6.1	22.5 ± 16.2	212 (7.2)	15 (7.1)	187/25 (88.2)	76.6 ± 27.0	128.7 ± 27.0	9 (4.2)
2014	80.7 ± 6.0	21.0 ± 15.0	297 (10.1)	125 (42.1)	252/45 (84.8)	78.2 ± 31.4	143.7 ± 60.2	13 (4.4)
2015	80.7 ± 5.9	26.0 ± 17.9	361 (12.3)	318 (88.1)	323/38 (89.5)	96.9 ± 43.9	138.7 ± 49.8	14 (3.9)
2016	80.4 ± 6.0	24.6 ± 15.8	466 (15.8)	461 (98.9)	406/60 (87.1)	87.2 ± 35.2	121.7 ± 39.3	12 (2.6)
2017	81.0 ± 6.3	23.7 ± 16.1	627 (21.3)	627 (100)	579/48 (92.3)	71.5 ± 32.9	119.9 ± 46.5	21 (3.4)
2018	80.7 ± 6.1	21.0 ± 15.0	538 (18.3)	538 (100)	514/24 (95.5)	64.9 ± 27.4	106.1 ± 46.1	8 (1.5)
Overall	80.9 ± 6.1	23.4 ± 15.9	2946 (100)	2084 (70.7)	2625/321 (89.1)	78.7 ± 35.5	130.3 ± 56.0	108 (3.7)

Patient and procedural characteristics of TAVR from 2008 to 2018 and overall means/counts. Continuous variables are displayed as mean with standard deviation, categorical variables as *n* (%), unless specified differently

*TF* transfemoral, *TA* transapical, *TAVR* transcatheter aortic valve replacement, *LogES I* Logistic EuroScore I

### Clinical outcomes

The primary outcome of 30-day mortality occurred in 3.7% overall, more likely in TA vs. TF TAVR patients (7.5% vs. 3.2%; *p* < 0.0001). Secondary clinical outcomes according to VARC-2 criteria are additionally reported in Suppl. Table 2.

### Risk model performance for the prediction of 30-day mortality

Risk model characteristics are provided in Table 1 and in the Supplementary material.

#### Model discrimination

**Fig. 1:**
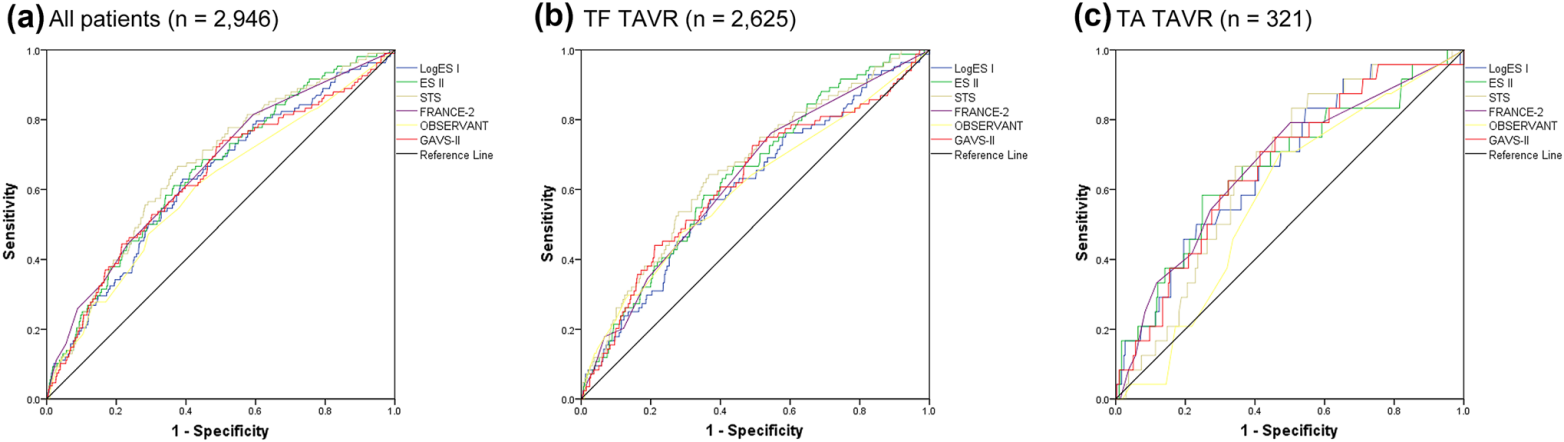
Risk model discrimination for the prediction of 30-day mortality. Model discrimination (ROC curves) of the six risk models for the prediction of 30-day mortality, for all studied patients **(a)**, patients with TF TAVR only **(b)** and patients with TA TAVR only **(c)**

Risk model discrimination performance indices are reported in Table 3A, Fig. 1 and Table 4: ROC analyses showed mediocre performance of all risk models, without significant differences between them. Numerically, STS PROM (*c*-index 0.67, 95% CI 0.62–0.72) performed best, followed by FRANCE-2 (*c*-index 0.66, 95% CI 0.60–0.71) and ES II (c-index 0.65, 95% CI 0.60–0.70), OBSERVANT performed worst (*c*-index 0.60, 95% CI 0.55–0.66). All risk models performed worse than in their original validation cohorts (Table 1), except for FRANCE-2 (*c*-index 0.66 vs. 0.59 original). STS PROM showed the most consistent performance across TF and TA TAVR subgroups (both *c*-indices 0.66), while OBSERVANT performed numerically worst in both.

**Table 3:** Risk model performance for the prediction of 30-day mortality risk in TAVR

	(A) Model discrimination: ROC analysis			(B) Model calibration: prediction of 30-day mortality risk		
Risk model	All patients (*n* = 2946)	TF TAVR (*n* = 2625)	TA TAVR (*n* = 321)	All patients (*n* = 2946)	TF TAVR (*n* = 2625)	TA TAVR (*n* = 321)
LogES I [7, 8]	0.63 (0.58–0.68)	0.61 (0.55–0.67)	0.67 (0.56–0.78)	23.4 ± 15.9 (1.1–95.0)	22.8 ± 15.7* (1.1–95.0)	28.0 ± 16.9* (2.5–88.4)
ES II [9]	0.65 (0.60–0.70)	0.64 (0.58–0.70)	0.67 (0.55–0.79)	7.5 ± 7.4 (0.5–78.1)	7.3 ± 7.2* (0.5–78.1)	9.3 ± 8.8* (1.0–56.1)
STS PROM [10]	0.67 (0.62–0.72)	0.66 (0.60–0.72)	0.66 (0.57–0.76)	6.2 ± 5.7 (0.5–69.3)	6.1 ± 5.6* (0.6–69.3)	7.5 ± 6.7* (0.6–58.1)
FRANCE-2 [15]	0.66 (0.60–0.71)	0.63 (0.57–0.69)	0.67 (0.55–0.79)	9.2 ± 6.8 (4.0–62.0)	8.3 ± 5.6* (4.0–55.0)	16.3 ± 10.7* (6.8–62.0)
OBSERVANT [14]	0.60 (0.55–0.66)	0.60 (0.54–0.67)	0.58 (0.47–0.68)	5.8 ± 5.0 (1.8–58.7)	5.7 ± 5.0 (1.8–58.7)	6.2 ± 4.9 (1.8–33.2)
GAVS-II [16]	0.63 (0.58–0.69)	0.62 (0.56–0.69)	0.67 (0.56–0.78)	9.4 ± 9.6 (1.0–89.8)	9.2 ± 9.1 (0.9–89.8)	10.9 ± 12.4 (1.1–80.8)

Summary of risk model performance indices for the prediction of 30-day mortality risk in TAVR. (A) Model discrimination, reported as *c*-indices (area-under-the-ROC-curve, Fig. 1) with 95% confidence intervals (in brackets); (B) model calibration reported as predicted mean 30-day mortality risk (in %) ± standard deviations (in %) and risk range (in brackets, in %)

*TF* transfemoral, *TA* transapical, *TAVR* transcatheter aortic valve replacement

*Statistically significant differences between TF and TA TAVR groups

**Table 4:** Risk model discrimination stratified by time period and device type

Risk model	2008–2010 (*n* = 139)	2011–2013 (*n* = 518)	2014–2016 (*n* = 1124)	2017–2018 (*n* = 1165)	Old-generation TAVR device (*n* = 862)	New-generation TAVR device (*n* = 2084)	Overall (*n* = 2946)
LogES I [7, 8]	0.67 (0.50–0.85)	0.59 (0.48–0.70)	0.68 (0.59–0.77)	0.57 (0.47–0.68)	0.63 (0.55–0.71)	0.63 (0.56–0.71)	0.63 (0.58–0.68)
ES II [9]	0.65 (0.46–0.83)	0.57 (0.48–0.67)	0.70 (0.61–0.78)	0.65 (0.55–0.74)	0.63 (0.56–0.71)	0.66 (0.59–0.73)	0.65 (0.60–0.70)
STS PROM [10]	0.53 (0.32–0.74)	0.64 (0.56–0.73)	0.67 (0.58–0.76)	0.64 (0.54–0.74)	0.63 (0.56–0.70)	0.65 (0.57–0.72)	0.67 (0.62–0.72)
FRANCE-2 [15]	0.63 (0.49–0.76)	0.63 (0.51–0.74)	0.66 (0.57–0.75)	0.66 (0.57–0.76)	0.61 (0.53–0.69)	0.68 (0.61–0.76)	0.66 (0.60–0.71)
OBSERVANT [14]	0.61 (0.41–0.82)	0.53 (0.41–0.65)	0.68 (0.60–0.76)	0.57 (0.47–0.68	0.57 (0.49–0.66)	0.63 (0.56–0.70)	0.60 (0.55–0.66)
GAVS-II [16]	0.58 (0.37–0.79)	0.54 (0.42–0.66)	0.68 (0.60–0.77)	0.66 (0.57–0.76)	0.59 (0.50–0.68)	0.67 (0.60–0.74)	0.63 (0.58–0.69)

Risk model discrimination performance across the study period from 2008 to 2018, stratified by time period and device type, in comparison to the overall mean, displayed as c-indices (area-under-the-ROC-curve) with 95% confidence intervals (in brackets)

*TAVR* transcatheter aortic valve replacement

The stratified analyses for time period and for new vs. old generation devices (Table 4) showed no significant differences and also no visible performance trend in either risk model over the years.

#### Model calibration

Mean predicted mortality risk (Table 3B) exceeded observed mortality in all models, ranging from 5.8 ± 5.0% (OBSERVANT) to 23.4 ± 15.9 (LogES I). Predictions were significantly higher in the TA TAVR subgroup for LogES I, ES II, STS PROM and FRANCE-2, while the OBSERVANT and GAVS-II models showed no differences between subgroups.

**Fig. 2:**
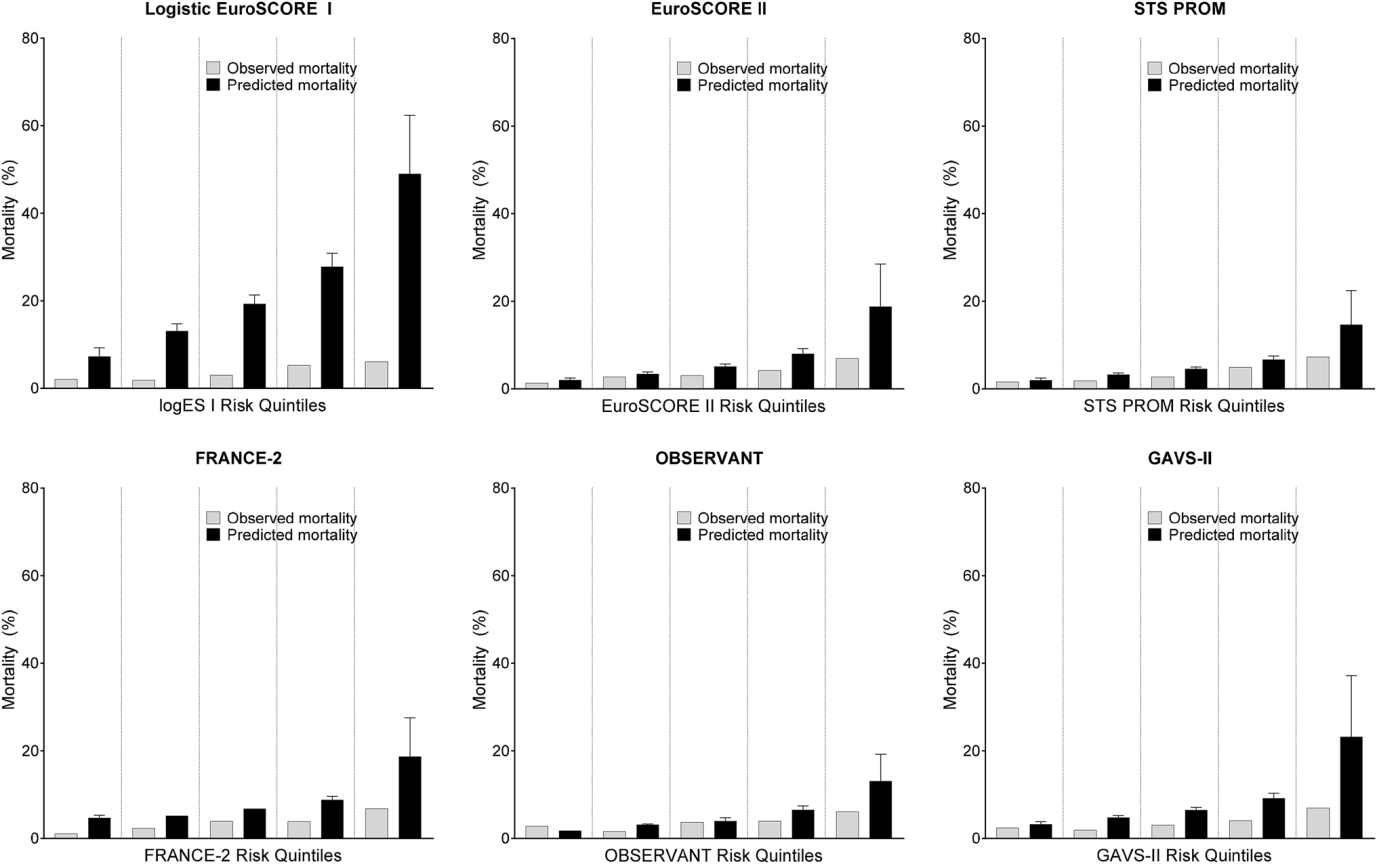
Risk model calibration for the prediction of 30-day mortality. Graphical model calibration comparison of the six risk models, stratified into risk quintiles for observed vs. predicted 30-day mortality

Graphical analysis of calibration is displayed in Fig. 2. Overestimation of mortality risk was especially pronounced in high-risk patients, while the lower risk quintiles were more adequately calibrated (e.g. STS PROM, ES II, OBSERVANT models). The classical LogES I surgical model grossly overestimated mortality in all risk strata.

Results of formal statistical testing for goodness-of-fit are displayed in Suppl. Table 3: STS PROM and GAVS-II showed significant Hosmer–Lemeshow *p *values (= inadequate calibration) of the respective logistic regression models for the whole population, GAVS-II also across TF and TA TAVR subgroups. OBSERVANT could not be calculated due to lack of events in some risk groups.

## Discussion

The main results of this comparative external validation study of six risk models for the prediction of 30-day mortality in patients undergoing TAVR at two German high-volume Heart Centers from 2008 to 2018 are (1) surgical and TAVR-specific risk models showed similarly mediocre discrimination performance (*c*-indices 0.60–0.67); (2) all models overestimated 30-day mortality risk and were poorly calibrated, especially in high-risk patients; (3) no significant influence of time of procedure or device type on the results could be found.

Patient risk stratification for coronary procedures is well established—ranging from the elective setting to acute coronary syndromes [22–24]—but the risk of TAVR procedures is thus far considerably less predictable: Patients suffer from a multitude of interdependent comorbid conditions, especially *frailty* as an essential risk factor is difficult to classify [25, 26]. The expansion of TAVR from highest risk/inoperable patients into intermediate- and lower risk groups introduces bias. On the procedural side, there are clear associations of center volume [27], operator experience [28], and choice of approach (TA vs. TF) [29] with clinical outcomes.

Classical surgical risk models (LogES I, STS PROM, ES II) have well-known limitations in TAVR [13]. LogES I is not recommended for use in TAVR anymore (1), we included the model for historical comparisons. National TAVR-registries have provided platforms for the development of TAVR-specific risk models [14–16], which were mostly developed in the “early years” of TAVR (Table 1). Resulting limitations in their performance have already been seen when applied outside of their original populations [30, 31], external validation studies with a similar design to our study were performed in the United Kingdom [12] and in the Netherlands [18]—and also retrieved disappointing results.

This analysis from the German Transregio Aortic Diseases cohort elucidates risk prediction in the whole era of TAVR, which has developed from an experimental procedure in 2008 to a routine and high-volume alternative to surgical aortic valve replacement in 2018. With steadily increasing operator experience and new-generation devices, procedure counts have dramatically increased and complication rates declined over the study period. The TF approach has become the routine access route.

Surgical risk models performed similar to expectations [13]: Discrimination analysis essentially showed similar performance of STS PROM [10], LogES I [7, 8] and ES II [9] models. None could even remotely match discrimination performance in their original surgical patient cohorts. Calibration analysis underlined the general overestimation of 30-day mortality risk—most pronounced in the LogES I model. Surgical models are thus confirmed to have severe limitations to judge TAVR risk.

However, dedicated TAVR-specific models also disappointed: FRANCE-2 (overall c-index 0.66, 95% CI 0.60–0.71) was better than in the original validation cohort (*c*-index 0.59 [15]) and in the UK (*c*-index 0.62 [12]) and Netherlands (*c*-index 0.63 [18]) external validation studies, but not superior to surgical models and slightly worse than in an Israeli external validation study (*c*-index 0.71 [30]). The model considerably overestimated risk (Fig. 2) in our patients. The GAVS-II model [16] derived from the German Aortic Valve Registry was expected to be most adapted to German TAVR conditions, but it performed similar to the other models in the overall cohort (*c*-index 0.63) and in TF and TA subgroups and could not meet the performance in the development cohort (*c*-index 0.74), similar to the Dutch external validation study [18]. Numerically, its discrimination improved over time and in new vs. old generation devices (Table 4). The model also overestimated mortality (expected: 9.4%; Fig. 2). Reasons for the lower-than-expected performance may lie in the GAVS-II model being developed in 55% surgical/45% TAVR patients from 2011–2012 (Table 1), which significantly differed in age (74 vs. 81 years) and comorbidities from our population [16]. The OBSERVANT model [14] discriminated numerically worst in the overall cohort and in TF/TA subgroups, and could not match performance in the original population (*c*-index 0.71), which confirms findings in other external validation studies [12, 18, 30]. However—of all analyzed models—its mean mortality prediction (5.8%) came closest to observed events (3.7%).

Taken together, this analysis clarifies that risk prediction in TAVR is still an unsolved issue: all tested models are severely limited in their performance, and dedicated TAVR-specific models are not superior to decade-old surgical scores. Using any of these models for risk stratification is better than a coin-flip—but with ample room for improvement. A recently published analysis from the Netherlands [18] found similar results to the UK study [12] and to our results, which supports the validity of these findings across Europe. With TAVR evermore becoming a routine procedure and 30-day mortality rates reaching all-time-lows in high-volume Heart Centers, a re-calibration of existing risk models or a new development from growing registries is thus mandatory to improve accuracy [32, 33]. High-quality TAVR databases should enable us to produce risk models with performance comparable to surgical procedures or acute coronary syndrome. Incorporation of functional status/frailty assessment as important predictive factors may additionally improve model accuracy in the TAVR setting [34, 35].

### Study limitations

All conclusions from our study are limited to risk model performance in German—or at best European—TAVR patients, findings might be different in other patient populations and procedural conditions. Bias may originate from retrospective calculation of FRANCE-2, OBSERVANT and GAVS-II risk models. While data quality in our prospective databases was high at all times, there is unavoidable risk for bias by changes in clinical practice and adverse event rates from 2008 to 2018: We accounted for this with sub-analyses of patients stratified by time of procedure and device generations (Table 4); however, their statistical power is limited.

## Conclusions

Three surgical as well as three TAVR-specific risk scoring models showed mediocre performance in prediction of 30-day mortality risk for TAVR in the German Rhine Transregio Aortic Diseases cohort. Development of new or updated risk models is necessary to improve risk stratification.

## Electronic supplementary material

Below is the link to the electronic supplementary material.

Supplementary file1 (DOCX 61 kb)

## Data Availability

Not applicable.

## Abbreviations

ES II: EuroSCORE II
EuroSCORE: European System for Cardiac Operative Risk Evaluation
GAVS-II: German Aortic Valve Score (2nd update)
LogES I: Logistic EuroSCORE I
OBSERVANT: Observational study of appropriateness, efficacy and effectiveness of AVR-TAVR procedures for the treatment of severe symptomatic aortic stenosis
STS (PROM): Society of Thoracic Surgeons Predicted Risk of Mortality
TAVR: Transcatheter aortic valve replacement
