# Supplementary material for: Risk modeling in transcatheter aortic valve replacement remains unsolved: an external validation study in 2946 German patients
Source: Clin Res Cardiol. 2020 Aug 26;110(3):368–76. doi: 10.1007/s00392-020-01731-9 (PMC7907023; doi:10.1007/s00392-020-01731-9)
Supplement: Supplementary file 1 — Supplementary file1 (DOCX 61 kb) [file 392_2020_1731_MOESM1_ESM.docx]

**Supplementary Online Material**

**Suppl. Table 1 – Patient characteristics and prosthetic TAVR devices**

| **Patient characteristics** | **All patients**  (n=2,946) | **TF TAVR**  (n=2,625) | **TA TAVR**  (n=321) |  |
| --- | --- | --- | --- | --- |
| Age (years) | 80.9 ± 6.1 | 81.1 ± 6.0 * | 78.7 ± 6.5 * |  |
| Female | 1491 (50.6) | 1366 (52.0) * | 125 (38.9) * |  |
| BMI | 26.8 ± 5.5 | 26.9 ± 5.6 | 26.3 ± 4.4 |  |
| Coronary artery disease | 1979 (67.2) | 1720 (65.5) * | 259 (80.7) * |  |
| Previous PCI | 1149 (39.0) | 1013 (38.6) | 136 (42.4) |  |
| Previous BAV | 104 (3.5) | 91 (3.5) | 13 (4.0) |  |
| Previous CABG | 437 (14.8) | 345 (13.1) * | 92 (28.7) * |  |
| Previous valve surgery | 62 (2.1) | 58 (2.2) | 4 (1.2) |  |
| Diabetes mellitus | 889 (30.2) | 778 (29.6) | 111 (34.6) |  |
| Arterial hypertension | 2676 (90.8) | 2367 (90.2) * | 309 (96.3) * |  |
| Pulmonary hypertension | 1577 (53.5) | 1392 (53.0) | 185 (57.6) |  |
| Peripheral artery disease | 980 (33.3) | 758 (28.9) * | 222 (69.2) * |  |
| Cerebrovascular disease | 493 (16.7) | 389 (14.8) * | 104 (32.4) * |  |
| COPD | 751 (25.5) | 637 (24.3) * | 114 (35.5) * |  |
| **Functional characteristics** | | | | |
| AV area (cm^2^) | 0.74 ± 0.2 | 0.74 ± 0.2 | 0.76 ± 0.2 |  |
| AV dPmax (mmHg) | 66.0 ± 24.8 | 66.8 ± 24.8 * | 59.8 ± 24.4 * |  |
| AV dPmean (mmHg) | 39.4 ± 15.8 | 39.8 ± 15.7 * | 36.0 ± 16.3 * |  |
| NYHA III and IV | 2443 (82.9) | 2201 (83.8) * | 242 (75.4) * |  |
| **Prosthetic valve type** | | | | |
| Medtronic CoreValve | 679 (25.9) | 679 (25.9) | - |  |
| Medtronic Evolut R | 932 (35.5) | 932 (35.5) | - |  |
| Medtronic Evolut R Pro | 141 (5.4) | 141 (5.4) | - |  |
| Edwards Sapien XT | 151 (5.1) | 52 (2.0) | 99 (30.8) |  |
| Edwards Sapien 3 | 811 (27.5) | 613 (23.4) | 198 (61.7) |  |
| Boston Lotus | 141 (5.4) | 141 (5.4) | - |  |
| Other | 93 (3.2) | 69 (2.6) | 24 (7.5) |  |

Suppl. Table 1: Patient baseline characteristics, functional aortic valve characteristics and prosthetic TAVR device details, for the whole cohort and for subgroups undergoing TF and TA TAVR. Values are mean ± standard deviation or n (%). * indicate statistically significant differences between TF and TA TAVR subgroups.

TF = transfemoral, TA = transapical, TAVR = transcatheter aortic valve replacement, BMI = body mass index, PCI = percutaneous coronary intervention, BAV = balloon aortic valvuloplasty, CABG = coronary artery bypass graft, COPD = chronic obstructive pulmonary disease, AV = aortic valve, NYHA = New York Heart Association classification.

**Suppl. Table 2 – Clinical outcomes**

| **Primary clinical outcome** | **All patients**  **(n=2,946)** | **TF TAVR**  **(n=2,625)** | **TA TAVR**  **(n=321)** | |
| --- | --- | --- | --- | --- |
| 30-day mortality | 108 (3.7) | 84 (3.2) * | 24 (7.5) * | |
| **Secondary clinical outcomes (in-hospital)** | | | |  |
| CPR | 110 (3.7) | 87 (3.3) * | 23 (7.2) * | |
| Conversion to surgery | 13 (0.4) | 10 (0.4) | 3 (0.9) | |
| Major bleeding | 112 (3.8) | 90 (3.4) * | 22 (6.9) * | |
| Major vascular complications | 127 (4.3) | 106 (4.0) * | 21 (6.5) * | |
| Stroke | 78 (2.7) | 65 (2.5) | 13 (4.1) | |
| Sepsis | 148 (5.0) | 124 (4.7) * | 24 (7.5) * | |
| AKI (Stage I-III) | 429 (14.6) | 350 (13.3) * | 79 (24.6) * | |
| New pacemaker | 427 (14.5) | 399 (15.2) * | 28 (8.7) * | |

Suppl. Table 2: Clinical outcomes for all patients and for subgroups undergoing TF and TA TAVR, respectively. Values are n (%). * indicate statistically significant differences between TF and TA TAVR subgroups.

TF = transfemoral, TA = transapical, TAVR = transcatheter aortic valve replacement, CPR = cardiopulmonary resuscitation, AKI = acute kidney injury.

**Suppl. Table 3 – Hosmer-Lemeshow test (goodness-of-fit)**

| **Risk Model** | **All patients**  **(n=2,946)** | **TF TAVR**  **(n=2,625)** | **TA TAVR**  **(n=321)** |
| --- | --- | --- | --- |
| LogES I (1, 2) | 0.24 | 0.24 | 0.93 |
| ES II (3) | 0.28 | 0.27 | 0.44 |
| STS PROM (4) | 0.02 * | 0.34 | 0.30 |
| FRANCE-2 (5) | 0.10 | 0.16 | 0.47 |
| OBSERVANT (6) | n/a | n/a | n/a |
| GAVS-II (7) | 0.048 * | 0.01 * | 0.03 * |

Suppl. Table 3: P-values of the Hosmer-Lemeshow test, describing goodness-of-fit of each logistic regression risk model for prediction of 30-day mortality. * indicates statistical significance.

**Suppl. material – Risk model parameter overview**

Logistic EuroSCORE I

Age, gender, chronic pulmonary disease, extracardiac arteriopathy, neurological dysfunction, previous cardiac surgery, creatinine >200µmol/L, active endocarditis, critical preoperative state, unstable angina, lv function, recent MI, pulmonary hypertension, emergency, other than isolated CABG, surgery on thoracic aorta, post infarct septal rupture

EuroSCORE II

Age, gender, renal impairment, extracardiac arteriopathy, poor mobility, previous cardiac surgery, chronic lung disease, active endocarditis, critical preoperative state, diabetes on insulin, NYHA class, CCS class 4 angina, lv function, recent MI, pulmonary hypertension, urgency, weight of the intervention, surgery on thoracic aorta

STS PROM

Age, gender, race, weight, height, haematocrit, WBC count, platelet count, creatinine, dialysis, hypertension, immunocompromise present, peripheral artery disease, cerebrovascular disease, mediastinal radiation, cancer within 5 years, family history of premature CAD, sleep apnea, liver disease, unresponsive state, syncope, diabetes, endocarditis, chronic lung disease, illicit drug use, alcohol use, pneumonia, tobacco use, home oxygen, previous cardiac interventions, MI, heart failure timing, NYHA classification, cardiogenic shock, atrial fibrillation/flutter, second/third degree heart block, sick sinus, ventricular tachycardia/fibrillation, iv inotropes, ADP inhibitor, ACE/ARB, steroids, GPIIb/IIIa inhibitor, resuscitation, number of diseased vessels, left main stenosis, ejection fraction, LAD stenosis, aortic stenosis, mitral stenosis, aortic insufficiency, mitral insufficiency, tricuspid insufficiency, AV disease etiology, incidence of cardiac surgery, surgery status, IABP, catheter-based assist device, ECMO

FRANCE-2

Age, body-mass-index, NYHA class IV, pulmonary oedema, pulmonary hypertension, critical state, respiratory insufficiency, dialysis, transfemoral/transapical/subclavian approach

OBSERVANT

Glomerular filtration rate, critical state, pulmonary hypertension, diabetes mellitus, NYHA class IV, prior ballon aortic valvuloplasty, left ventricular ejection fraction <40%

GAVS-II

Gender, age, body-mass-index, NYHA class IV, CCS class III/IV, cardiogenic shock, cardiopulmonary resuscitation, pulmonary hypertension, sinus rhythm, ASA class 4/5, left ventricular ejection fraction <30%, coronary artery disease (number of diseased vessels), infectious endocarditis/septic condition, diabetes mellitus, arterial vessel disease, preoperative hemodialysis/creatinine >2.3 mg/dl, mechanical circulatory support, Redo surgery (heart/thoracic aorta)

**Supplementary References**

1. Roques F, Michel P, Goldstone AR, Nashef SA. The logistic EuroSCORE. Eur Heart J. 2003;24(9):881-2.

2. Nashef SA, Roques F, Michel P, Gauducheau E, Lemeshow S, Salamon R. European system for cardiac operative risk evaluation (EuroSCORE). Eur J Cardiothorac Surg. 1999;16(1):9-13.

3. Nashef SA, Roques F, Sharples LD, Nilsson J, Smith C, Goldstone AR, et al. EuroSCORE II. Eur J Cardiothorac Surg. 2012;41(4):734-44; discussion 44-5.

4. O'Brien SM, Shahian DM, Filardo G, Ferraris VA, Haan CK, Rich JB, et al. The Society of Thoracic Surgeons 2008 cardiac surgery risk models: part 2--isolated valve surgery. Ann Thorac Surg. 2009;88(1 Suppl):S23-42.

5. Iung B, Laouenan C, Himbert D, Eltchaninoff H, Chevreul K, Donzeau-Gouge P, et al. Predictive factors of early mortality after transcatheter aortic valve implantation: individual risk assessment using a simple score. Heart (British Cardiac Society). 2014;100(13):1016-23.

6. Capodanno D, Barbanti M, Tamburino C, D'Errigo P, Ranucci M, Santoro G, et al. A simple risk tool (the OBSERVANT score) for prediction of 30-day mortality after transcatheter aortic valve replacement. The American journal of cardiology. 2014;113(11):1851-8.

7. Schiller W, Barnewold L, Kazmaier T, Beckmann A, Masseli F, Welz A, et al. The German Aortic Valve Score II. Eur J Cardiothorac Surg. 2017;52(5):881-7.
